# Treatment of Bilateral Varicocele and Other Scrotal Comorbidities Using a Single Scrotal Access: Our Experience on 34 Patients

**DOI:** 10.1155/2014/403603

**Published:** 2014-07-23

**Authors:** F. Iacono, A. Ruffo, D. Prezioso, G. Romeo, E. Illiano, G. Di Lauro, L. Romis, S. Sansalone

**Affiliations:** ^1^Department of Urology, Federico II University, Via S. Pansini 5, 80100 Naples, Italy; ^2^Department of Urology, Hospital Santa Maria delle grazie, Via Domiziana, Località la Schiana, Pozzuoli, 80078 Naples, Italy; ^3^Department of Urology, Tor Vergata University, Viale Oxford 81, 00133 Rome, Italy

## Abstract

*Introduction*. Varicocele is the main cause of infertility in male and the most correctable cause of it too. In this study, we present our experience on 34 patients affected by bilateral varicocele and other scrotal comorbidities treated underwent surgery with a scrotal access. *Materials and Methods*. 34 patients were enrolled with clinical palpable and infraclinical (ultrasonic doppler scanning) bilateral varicocele and other comorbidities like right hydrocele, left hydrocele, bilateral hydrocele, and epididymal cyst. They all underwent scrotal bilateral varicocelectomy under local anesthesia. *Results and Discussion*. At 6 months, no other complications were reported. No case of testicular atrophy was observed. None had recurrence of varicocele. All scrotal comorbidities were treated as well. *Conclusion*. Scrotal access with local anesthesia is a safe and useful technique to treat patients with bilateral varicocele and other scrotal comorbidities.

## 1. Introduction

Varicocele is a common abnormality with the following andrological implications: failure of ipsilateral testicular growth and development, symptoms of pain and discomfort, male infertility. It is commonly believed that this condition may begin with the onset of puberty, at around the age of 15 [1].

Most varicoceles are left-sided, and the left-sided predominance is explained by turbulent venous flow related to the right angle insertion of the left testicular vein into the left renal vein [2]. Varicocele is a physical abnormality present in 11.7% of men with normal semen analysis and in 25.4% of men with abnormal semen [3].

Varicoceles are recognized as the most common surgically correctable cause of male infertility, but the exact mechanism of varicocele-induced impairment of spermatogenesis remains a matter of debate. The exact association between reduced male fertility and varicocele is unknown, but a meta-analysis showed that semen improvement is usually observed after surgical correction [4].

Varicocele is associated with increased sperm DNA damage, and this sperm pathology may be secondary to varicocele-mediated oxidative stress. Varicocelectomy can reverse this sperm DNA damage, as shown in several studies [5].

Physical examinations and scrotal ultrasounds remain the most commonly used methods. Varicocele is graded at the time of the initial physical examination according to the Dublin grading system (I–III) [6]. Surgical correction of varicocele improves their fertility potential [7]. Several surgical approaches to varicocelectomy exist, each with its own advantages and drawbacks: varicocele embolization, the traditional inguinal (Ivanissevich), or high retroperitoneal (Palomo) approaches, laparoscopic repair and microsurgical repair* via* an inguinal, or subinguinal incision.

Complications of varicocele repair include hydrocele formation, persistence or recurrence of the varicocele, and rarely testicular atrophy [8].

Although no specific recommendations exist as to the optimal surgical technique for varicocelectomy, the use of magnification to preserve lymphatics and testicular arteries is recommended.

We strongly believe that microsurgical varicocelectomy is the gold-standard technique for varicocelectomy in both adults and adolescents, due to lower postoperative recurrence and complication rates compared to other techniques [9].

However, surgery via a scrotal approach was not widespread due to the difficulty of preserving the arterial supply of the testis because the pampiniform plexus of veins encoils the testicular artery at the level of the scrotum. By the way we think that scrotal access is useful in the management of bilateral varicocelectomy in order to avoid two surgical incisions [10] and it can be a valid technique when there are other scrotal comorbidities to be treated.

## 2. Materials and Methods

We enrolled in our study, from February 2012 to March 2014, 34 adult patients with clinical palpable and infraclinical (ultrasonic doppler scanning) bilateral varicocele and other comorbidities like right hydrocele (6 pts), left hydrocele (8 pt), bilateral hydrocele (8 pts), and epididymal cyst (12 pts).

They underwent scrotal microsurgical bilateral varicocelectomy.

Varicocele has been classified into 4 stages. Before surgery all the patients underwent a complete physical examination, including supine and standing scrotal examination and a color doppler ultrasound examination.

**Figure 1:**
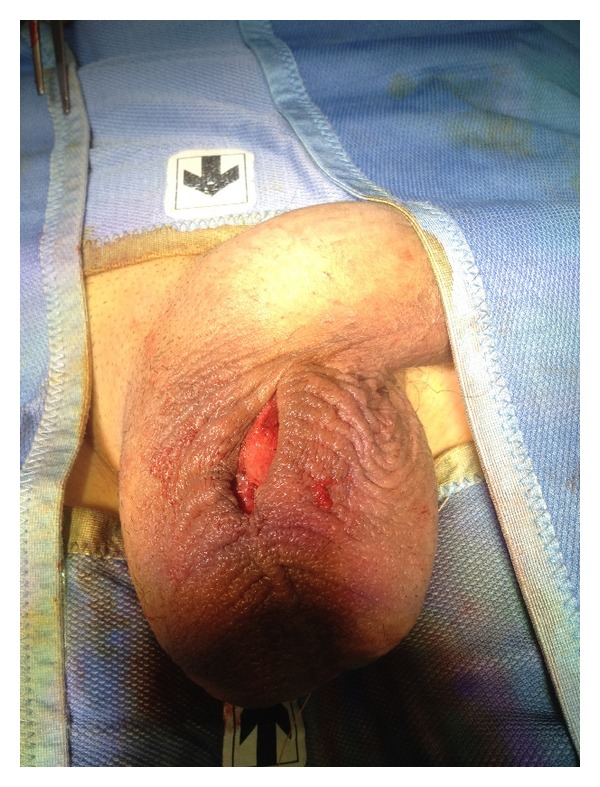
A median scrotal incision was done on rafe.

Under local anesthesia, a single incision was made on the median raphe, rather than two incisions at the root of the two hemiscrotums (Figure 1).

**Figure 2:**
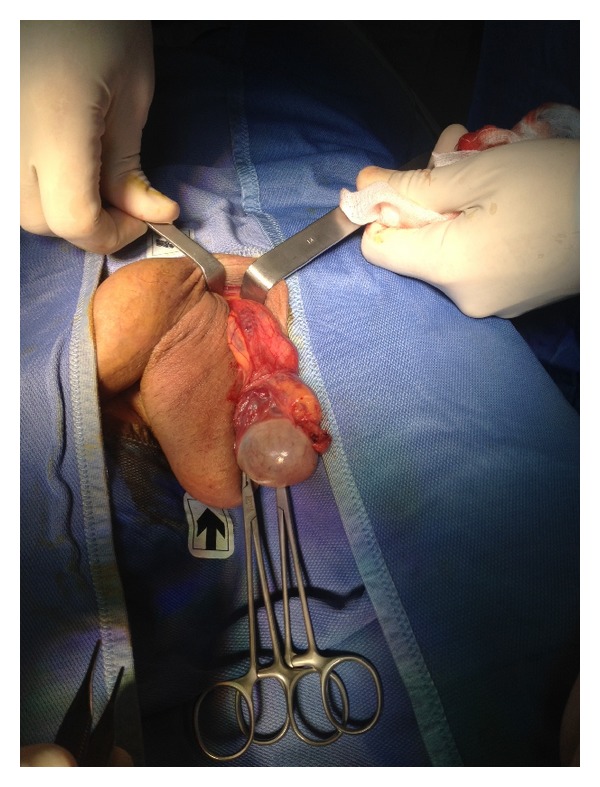
Exposure of left testis. A resection and eversion of the tunica vaginalis was performed in order to remove hydrocele.

Dartos fascia was open, and left testis was exposed by opening the tunica vaginalis in order to remove serous fluid given by a hydrocele. A resection and eversion of the tunica vaginalis was performed (Figure 2).

**Figure 3:**
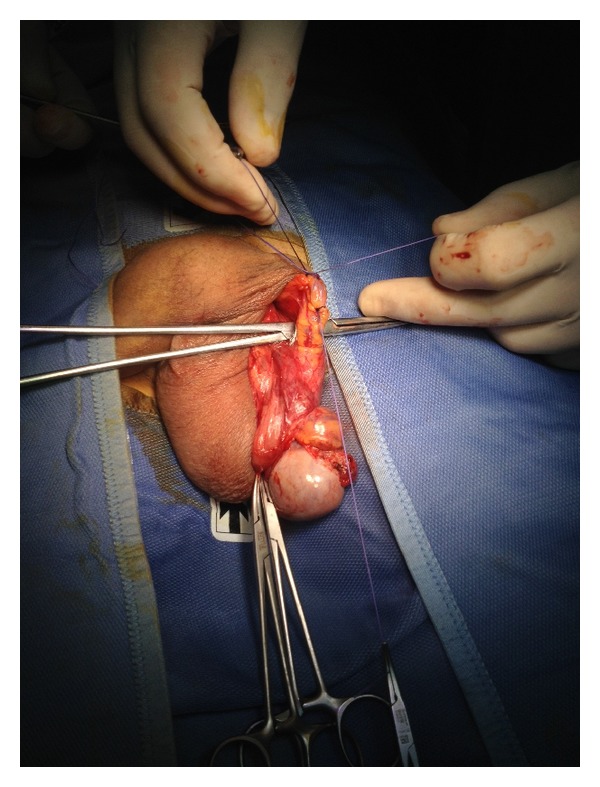
Ligation of the anterior spermatic venous plexus.

Using two Farabeuf retractors the left spermatic cord was exposed more proximally until the external inguinal ring and at this level the cremasteric and internal spermatic fascia were opened longitudinally with the exposure of the testicular vein. In this case we performed an en block ligation of the anterior spermatic venous plexus using an absorbable suture (2.0 vicryl) (Figure 3). In our opinion preserving the cremasteric and deferential arteries is enough to supply vascularization to the testis in cases where the testicular artery is damaged.

Cremasteric fascia was closed using an absorbable suture (5.0 vicryl).

**Figure 4:**
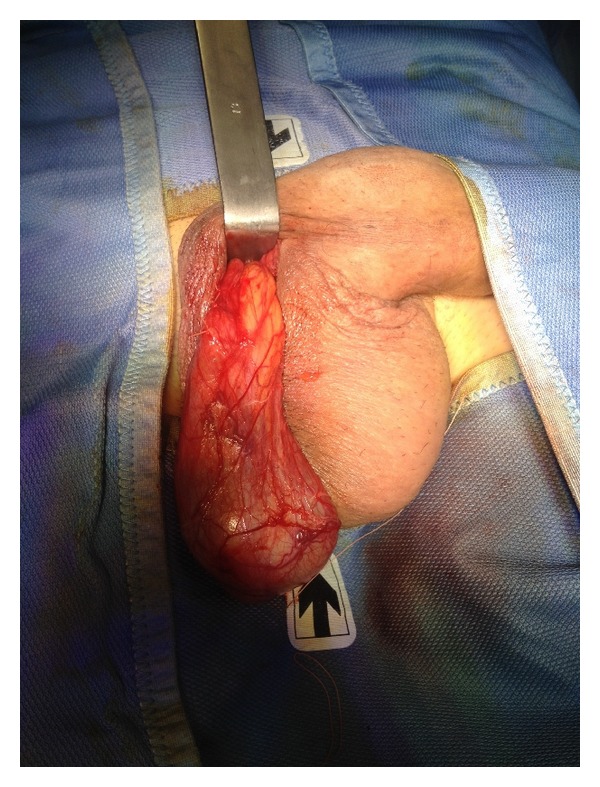
Exposure of the right testis.

**Figure 5:**
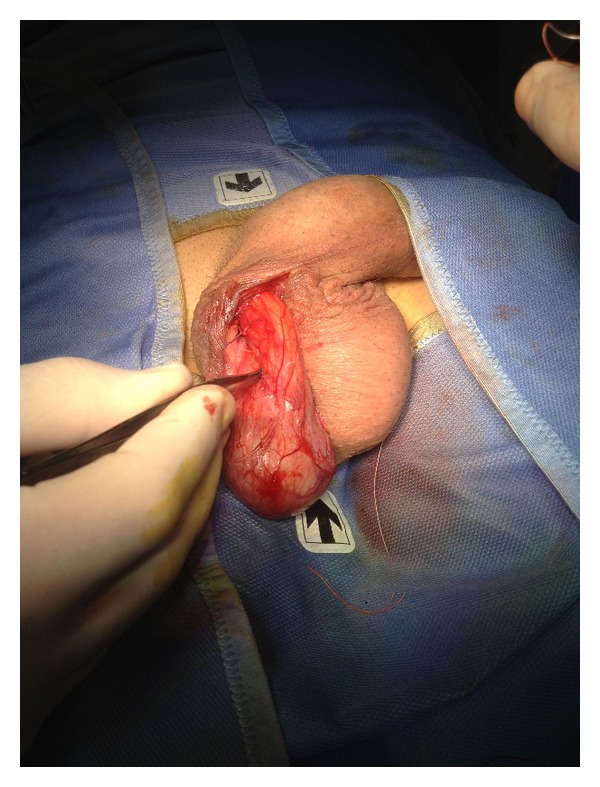
Exposure of the spermatic cord.

**Figure 6:**
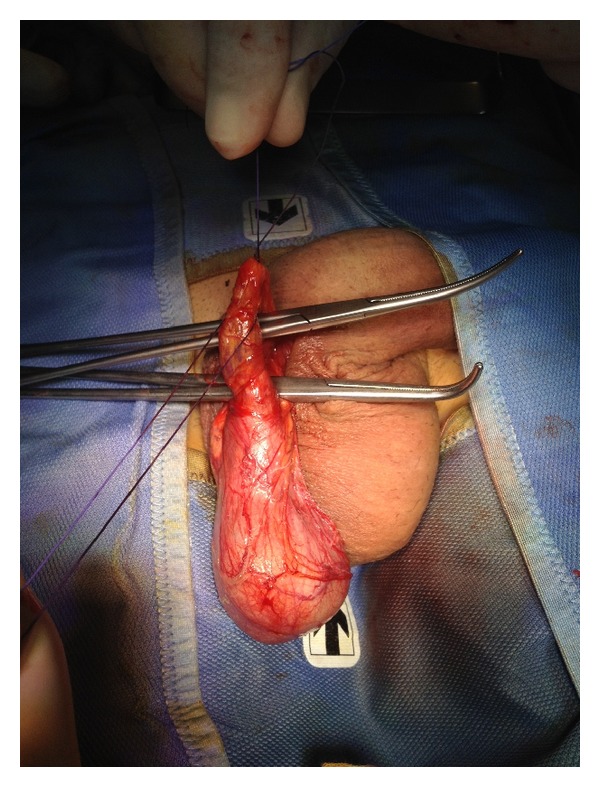
Ligation of the anterior spermatic venous plexus.

The same procedure was performed on the right testis (Figures 4, 5, and 6).

Dartos fascia was sutured using a continuous running suture using a 3.0 vicryl.

**Figure 7:**
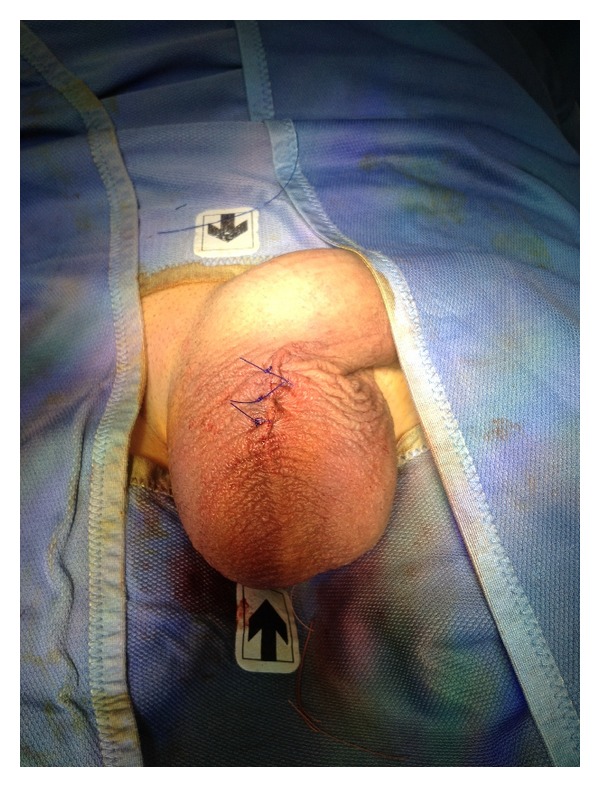
Closure of the scrotal incision with 3 nonabsorbable stiches.

Skin was closed using 3 stiches in nonabsorbable suture 3.0 Prolene (Figure 7).

Surgery for both testis lasted 30 minutes.

## 3. Results and Discussion

 All patients were evaluated at 1 week, at 3 and 6 months after the operation by means of physical examination, scrotal Doppler ultrasound, and sperm analysis.
 None of the patients reported pain at 3-month follow-up.
 Edema of the spermatic cord occurred in 12 pts with spontaneous regression at 3-month follow up, and in 2 pts contralateral hydrocele was observed.
 At 6 months no other complications were reported.
 No case of testicular atrophy was observed.
 None had recurrence of varicocele.
 In our opinion subinguinal varicocelectomy is the best approach for unilateral varicocele because it has the advantage of allowing the spermatic cord structures to be pulled up and out of the wound so that the testicular artery, lymphatics, and small periarterial veins may be more easily identified and preserved. In addition, subinguinal approach allows access to external spermatic and even gubernacular veins, which may bypass the spermatic cord and result in recurrence if not ligated.

In the early 1900s, an open scrotal approach was employed, involving the mass ligation and excision of the varicose plexus of veins. At the level of the scrotum, however, the pampiniform plexus of veins is intimately entwined with the coiled testicular artery. For many authors scrotal operations are to be avoided because damage to the arterial supply of the testis frequently results in testicular atrophy. For this reason, in the scrotal approach we expose the spermatic cord more proximally, at level of the external ring, in order to avoid any damage to the testis vascularization (Figure 2).

However, anatomic studies have proved that the diameter of the testicular artery is the main blood supply to the testis being greater than the diameter of the deferential artery and cremasteric artery combined [11].

By the way we believe that the deferential (vasal) artery and, if preserved, the cremasteric artery, will provide adequate blood supply to the testes to prevent atrophy.

## 4. Conclusions

In our opinion varicocele repair must be proposed in young adult men with impairment of seminal parameters. Patients with bilateral varicocele prefer a single incision. When the incision is made on the median raphe, no scars remain. In bilateral varicocele with other scrotal comorbidities the single approach reduces invasiveness and increases patient satisfaction.
